# Deep neural network models for identifying incident dementia using claims and EHR datasets

**DOI:** 10.1371/journal.pone.0236400

**Published:** 2020-09-24

**Authors:** Vijay S. Nori, Christopher A. Hane, Yezhou Sun, William H. Crown, Paul A. Bleicher

**Affiliations:** OptumLabs, Boston, Massachusetts, United States of America; Banner Alzheimer’s Institute, UNITED STATES

## Abstract

This study investigates the use of deep learning methods to improve the accuracy of a predictive model for dementia, and compares the performance to a traditional machine learning model. With sufficient accuracy the model can be deployed as a first round screening tool for clinical follow-up including neurological examination, neuropsychological testing, imaging and recruitment to clinical trials. Seven cohorts with two years of data, three to eight years prior to index date, and an incident cohort were created. Four trained models for each cohort, boosted trees, feed forward network, recurrent neural network and recurrent neural network with pre-trained weights, were constructed and their performance compared using validation and test data. The incident model had an AUC of 94.4% and F1 score of 54.1%. Eight years removed from index date the AUC and F1 scores were 80.7% and 25.6%, respectively. The results for the remaining cohorts were between these ranges. Deep learning models can result in significant improvement in performance but come at a cost in terms of run times and hardware requirements. The results of the model at index date indicate that this modeling can be effective at stratifying patients at risk of dementia. At this time, the inability to sustain this quality at longer lead times is more an issue of data availability and quality rather than one of algorithm choices.

## Introduction

Over 50 million people lived with dementia worldwide in 2019, and those numbers are expected to increase to 152 million by 2050 [1]. Within the United States, an estimated 5.8 million people of all ages are living with the most common form of dementia, Alzheimer’s disease (AD), and this number is expected by rise to 13.8 million by the year 2050 [2]. The total health care and long-term care costs associated with AD and related dementias were $277 billion in the US in 2018 [2]. Tools are needed to assist clinicians, public health workers, and epidemiologists in identifying individuals at risk for dementia and addressing this unfolding epidemic.

During the last few years predictive models have been published which help with identifying people with high risk of ADRD prior to the onset of the disease. These models can be broadly divided in two groups. In the first group authors select risk factors based on clinical input and quantify their effect using statistical models [3–9]. In the second group, which is much smaller than the first, Machine Learning (ML) data-driven models have been developed [10, 11] to identify and quantify the risk factors from large healthcare data resources. Recent work [12] on predicting dementia not including Mild Cognitive Impairment (MCI), 1 or 3 years prior to the onset of the disease using ML models trained on EMR data (diagnosis, medical notes, prescriptions) along with demographics (age, gender, race) showed an improved accuracy over previous work, and clinically relevant features. However, this article mentions using all medical records within 10 years prior to the index date which is not available in most systems or settings. While the body of work using ML models in this area is limited, they have been used extensively for identifying people at risk in a variety of other areas such as diabetes [13], chronic kidney disease [14], heart failure [15], cardiovascular disease [16], etc. The advantage of these methodologies is the ability to choose the most predictive out of tens of thousands of features without the need for clinical input, and create models which typically have better (or at least the same) accuracy than the curated models. For a condition like ADRD, the onset of which can impact not just the patient but the family [17], using predictive models to identify high-risk patients followed by a comprehensive screening test, can help in many ways including changes in lifestyle, better planning for the future, etc. Early and accurate identification of such individuals is also very important for clinical development of novel treatments. Unfortunately, patients are identified after they are already symptomatic and have already experienced significant neurodegeneration. Screening patients into high risk groups can facilitate development of programs that investigate causal relations to specific ADRD etiologies, and recruitment to clinical trials. While a clinician may be able to identify some important precursors for a condition, a machine learning approach is preferred when predictive risk models have to be built using temporal snapshots of healthcare events several years before the onset of the disease.

Recent advances in Deep Neural Network (DNN) methods, a subset of machine learning [18], have shown great promise in harnessing the power of large administrative claims and electronic health record (EHR) datasets and advanced computation to come up with more accurate healthcare predictive models. Rajkomar, *et al*. [19] developed deep learning models using EHR data for predicting tasks such as patient’s final discharge diagnosis and 30-day unplanned re-admission with a c-statistic of 0.9 and 0.76, respectively. Their models used sequences of events leading up to an index event as inputs to Long short-term memory (LSTM) recurrent neural networks [20] which are able to leverage the sequence and time between event information. Choi, *et al*. [21] developed a temporal predictive model using Gated Recurrent Units (GRU) [22] which use medical history (diagnosis, procedure, medication) and time between visits to predict diagnosis and medication orders, and time to next visit. Their results showed an 8% improvement in area under the curve (AUC) when compared with predictions from logistic regression models. In a later paper Choi et al [23] developed a neural attention model [24] again using GRUs which not only provides a score for the prediction model, but also interprets the score (using attention weights) by showing the importance of the features which contributed to it. Attention models have been successfully used in a variety of domains from Natural Language Processing to Computer Vision [25, 26]. Benefits of these models include managing performance degradation with increase in length of the input sequence which is a limitation of RNNs, but come with an additional cost of training more weights. Sequence lengths are not as important when predicting conditions such as Alzheimer’s multiple years from the onset of the disease where the amount of data available decreases rapidly. Kim *et al*. [27] used LSTM-based models to identify people who may be at risk for Alzheimer’s disease and all-cause dementia. While the AUC for their DL-based model on validation data is about 90%, they use data over nine years and in most cases very close to the index date, and use data elements including lifestyle not commonly available in most datasets.

In this paper a set of Deep Neural Network models have been developed to predict if a person may be at a risk for incident Alzheimer’s disease and related dementias, and mild cognitive impairment, 3 to 8 years before the onset of the disease. Models are based on Feed forward networks (multi-layer perceptrons), recurrent neural networks (RNN), RNNs with pre-trained code embeddings (a mapping of each code to vector of numbers), and these architectures have been modified to improve accuracy for a condition whose prevalence is highly correlated with age. Extensive grid searches have been performed to identify the best set of parameters for models. The results are compared with those obtained using gradient boosted trees using the approach described in Nori *et al*. [28].

## Materials and methods

### Datasets

This study used de-identified administrative claims and electronic health record (EHR) data from the OptumLabs^®^ Data Warehouse (OLDW) [29]. The database contains longitudinal health information on individuals, representing a diverse mixture of ages, ethnicities and geographical regions across the United States. The claims data in OLDW includes medical and pharmacy claims, laboratory results and enrollment records for commercial and Medicare Advantage enrollees. Clinical notes are available from a subset of EMR systems who choose to share this data and have been converted to structured data elements. Since this study involved analysis of pre-existing, de-identified data, it was exempt from Institutional Review Board approval [30]. The study dates from 1/1/2007 to 12/31/2017 coincide with the earliest date that EHR data is available, and the end of the latest year after the project was initiated. Patients are 45 years old or more on their confirmation date.

The study outcome was a binary variable indicating a new diagnosis of ADRD or MCI. Identification rules for this cases are described in Appendix A in S1 File. The index date for individuals (cases), whose diagnosis was confirmed using two events (1, 3 and 4) was set as the second of the two events (the first confirmed date with ADRD), whereas for criteria 2 and 5, it is the earliest of those events. To ensure that the identified individuals have incident rather than prevalent ADRD, a 24-month period of continuous enrollment was required without any of the above events before the index date.

The steps involved in creating the training, validation and test cohorts are presented in Appendix B in S1 File. Starting with the entire population in the database, the attrition flowchart shows the number of people who survive in each step leading up to the final cohorts. The facts collected during the two-year window include diagnoses (ICD-9 & 10), procedures (CPT-4), medication (generic name from pharmacy fills and prescriptions written) in the sequence in which they occurred. Codes for a hierarchical code map for diagnosis codes called Episode Treatment Groups [31] were added in addition to the original codes to handle sparsity issues. Demographic features age and gender, and a utilization feature, coded as the number of distinct encounters was also added.

Input to models is provided in one of two ways, as a matrix or as code sequences. For models which use a matrix, it is constructed as the number of distinct days on which a person (row) had a code (column). The extreme values in the matrix were limited (winsorized) at the 95^th^ percentile of values computed using the training data. All sparse columns were scaled using the maximum value for the column whereas the dense columns were normalized using mean and standard deviation. Models which use code sequences for a person, use 300 codes from the relevant 2-year data collection period which are closest to the index date, with no more than 10 occurrences of a code. This allows the training to proceed efficiently while not effecting the accuracy.

### Analytic methods

**Fig 1:**
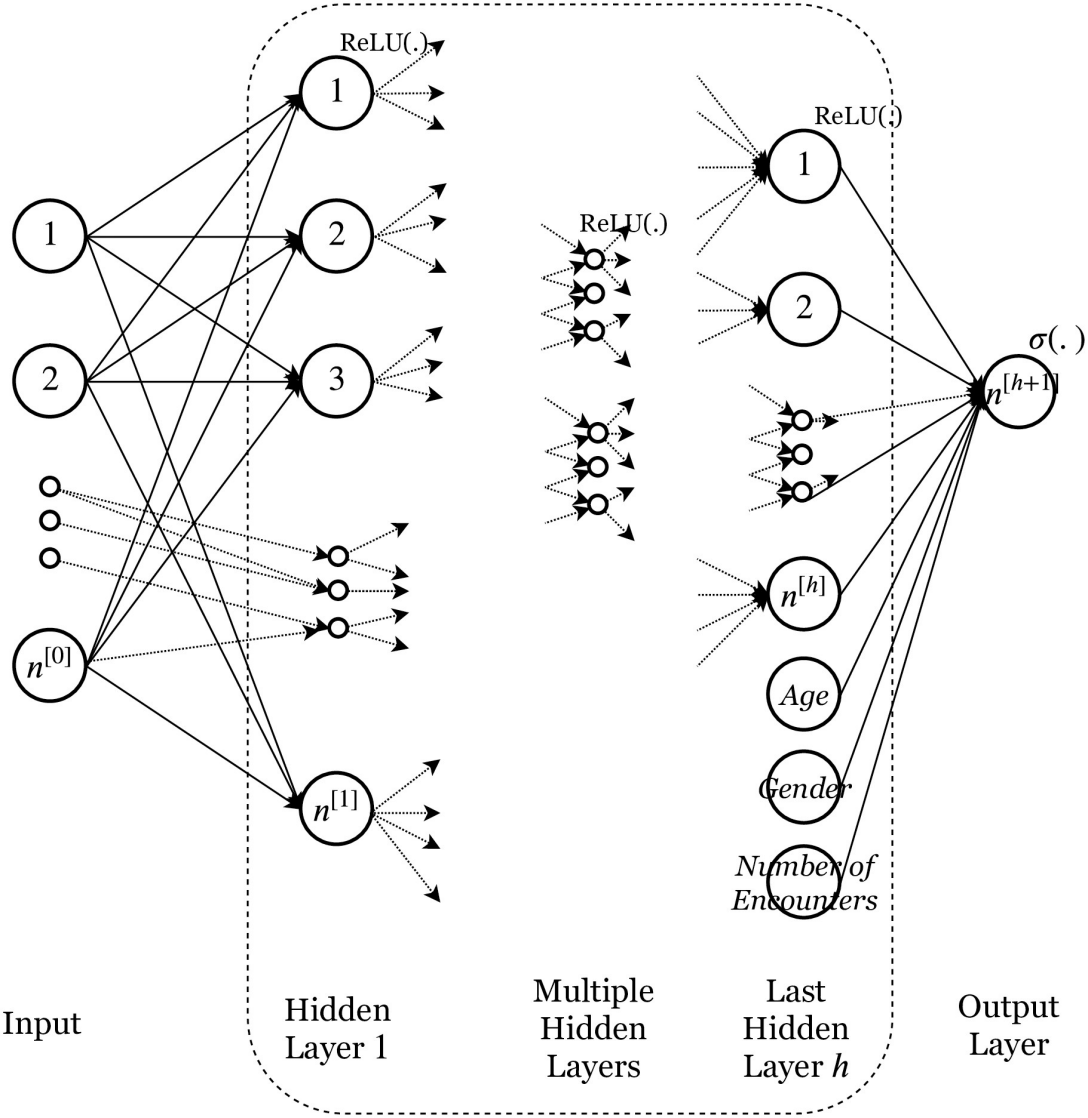
Feed forward network architecture.

Two different deep neural network architectures were used, one a feed forward network and the other based on a recurrent neural network. Fig 1 shows the architecture for the deep feed forward neural network with input in matrix form. The network has multiple hidden layers of different sizes, with the output of each of those layers passing through a Rectified Linear Unit (ReLU) activation function [32]. The output layer uses a sigmoid or logistic activation function. A dropout layer [33] used for regularization to prevent overfitting is used between each of the hidden layers and between the input and first hidden layer.

A key novelty in the architecture is to include the features age, gender and number of encounters, in the last hidden layer and connected directly with the output layer. This allows for each of these key features more influence because there isn’t a dropout layer between this and the output layer. The other weights get adjusted accordingly in the presence of these features. The network was compared against a network where age, gender and number of encounters were included with all the other features in the input layer. The modification with the three features in the last hidden layer performed better on all instances and hence is the only one presented here.

**Fig 2:**
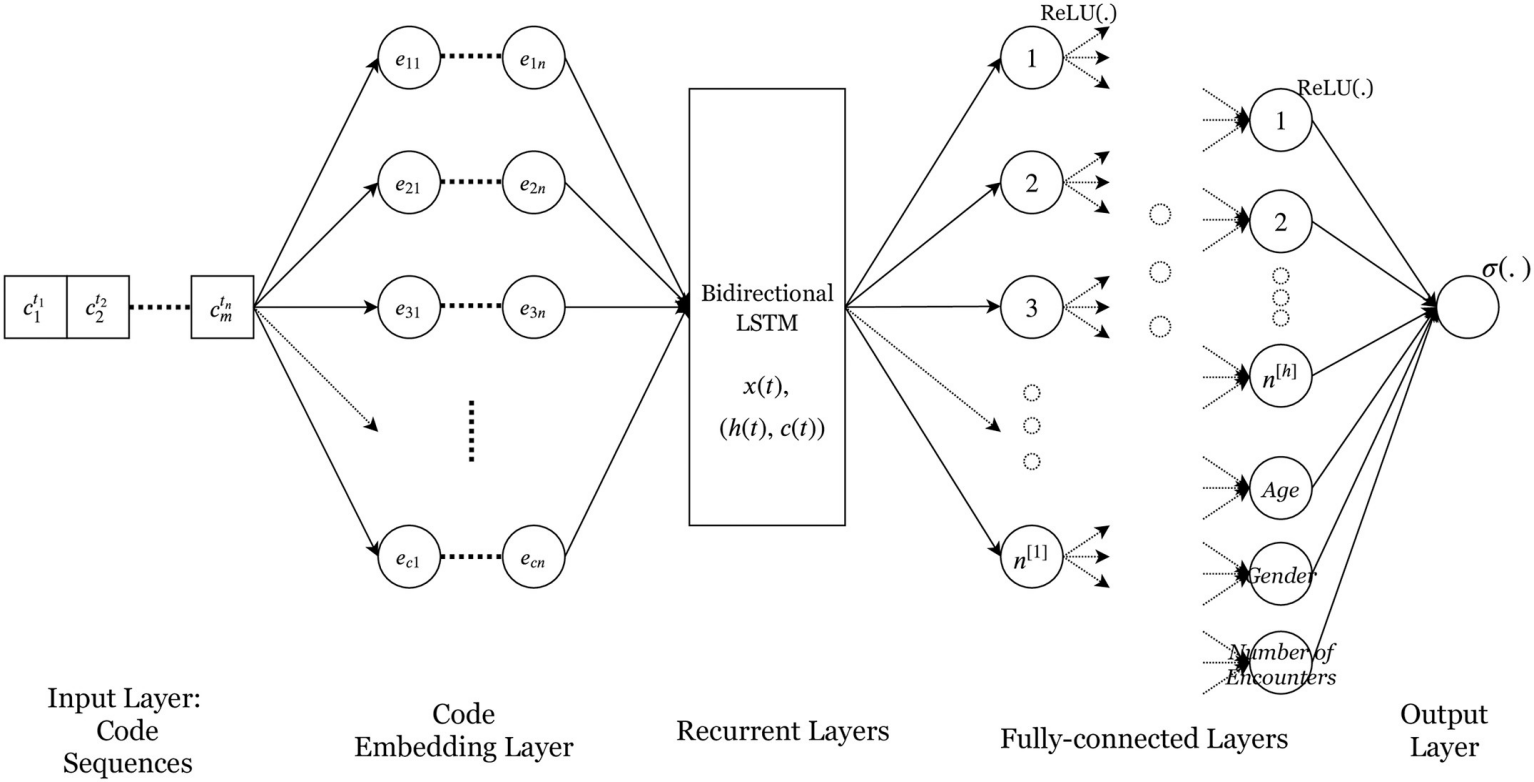
Recurrent neural network architecture.

Fig 2 shows the RNN-based architecture where the inputs are code sequences for each person in the cohort. This is passed through an embedding layer where an N-dimensional vector is estimated for each code. These vectors are passed in chronological order through a bidirectional LSTM which summarizes the codes from the past and from the future to create a more enriched hidden state. The information in the hidden state is then passed through a set of fully connected layers with ReLU activation and dropouts between the layers before the output layer with sigmoid activation. Multiple variations of the LSTMs, two LSTMs where the output of one is fed to the other, a single LSTM which only uses the past states and Gated Recurrent Units were tried before finally choosing the above architecture which consistently performed better than the others.

All the parameters, including the embedding vectors, of the RNN model are initialed with random numbers with the values updated after every epoch. A variant of this architecture where embedding vectors from another trained model is read in was also tried. The weights from the RNN model on incident cohort (year0) were saved and read in for training the other six models (year3,…, year8). The set of features which are in each of the models depend on the codes during the data collection period and can be different. So, not all the features in yearK have pre-trained embedding values in year0. Three different approaches were tried with the commonality of reading in the embeddings for features if they are available and setting them to zero if they are not present in year0: (i) freeze all the embedding values, (ii) use the input values as initial values and train them as before, (iii) freeze the embedding for available codes, and train those that aren’t available. The results for the best of these three is presented in the next section.

Training the deep neural networks involves architectural and parameter choices that finally determine the accuracy of the model. For a feed forward network architectural choices include the number of hidden layers, the size of each layer, and drop out probability. The parameter choices include name of the stochastic gradient descent optimization algorithm, learning rate, mini-batch size, number of epochs, and stopping conditions. Parameter choices for RNN networks include changing the size of the embedding vector, size of the hidden state in the LSTM, as well as all the choices for the fully connected layers and other parameters, which are the same as those for the feed forward network. While there is some literature on efficient methods for selecting hyperparameters [34, 35], these methods can be model specific and are very heuristic given that the search space is non-convex making it hard to optimize. The method preferred here is a grid search where a Cartesian product of all parameters is evaluated and the best combination is selected based on F1 score on validation data.

The Deep Neural Network models were run on a Dell DGX-1 Server with 8 nVidia Tesla V100 16GB GPUs using CUDA 10.1 drivers. The models were coded using PyTorch v1.2 [36] and grid search parameter sets were run in parallel across the GPUs. These models are compared against a baseline set of boosted tree models also optimized using grid search trained on Intel Xeon E5-2698s with 28 threads using LightGBM v2.3.1 [37].

## Results

### Cohort characteristics

Table 1 shows statistics for age on index date, gender and number of encounters (days) during data collection period for all the cohorts. The average age on index date increases monotonically from year0 to year8, as the cohorts have facts collection period away from the index date. The span during which continuous enrollment is required increases although the CE requirement is the same four years for all; for year3 the span is 5 years whereas for year8 it is 10years. The longer span requirement naturally leads to a higher average age although that effect is tempered by the fact that age in the database is truncated at 89 to protect privacy. The monotonic reduction in percentage of males is linked to the increase in age and that life expectancy of women is higher than that of men. The increase in number of encounters for cases from year8 to year0 is explained by the fact that they are progressing to a diagnosis and need more trips to their physician.

**Table 1:** Age, gender and number of encounter statistics for cohorts.

Feature	Type	year0	year3	year4	year5	year6	year7	year8
Age, years mean (sd)	Case	75.8 (10)	76.8 (9.8)	77 (9.7)	77.3 (9.7)	77.5 (9.7)	77.8 (9.6)	78.2 (9.3)
	Control	60.9 (10.8)	63.6 (10.4)	64.3 (10.3)	64.8 (10.2)	65.4 (10)	66 (9.9)	66.8 (9.8)
Gender, male N (%)	Case	147,621 (37.5)	54,906 (37.2)	38,574 (37.1)	25,714 (36.9)	16,174 (36.6)	9,248 (36.3)	4,125 (36.1)
	Control	5,060,258 (43.4)	989,495 (41.2)	634,054 (40.9)	392,646 (40.6)	232,967 (40.5)	127,122 (40.3)	55,056 (40.1)
Encounters mean (sd)	Case	35.1 (32)	27.9 (25.5)	26.7 (24.5)	25.4 (23.7)	24.3 (23)	23.5 (22.7)	23.1 (21.9)
	Control	21.1 (22.1)	18.3 (18.5)	17.6 (17.9)	16.9 (17.3)	16.3 (16.8)	16.3 (16.8)	16.7 (17.2)

The univariate odds ratios of some of the diagnosis, procedure and drug features out of thousands of features in the training cohorts with a prevalence of at least 1% in either the cases or controls are presented in Appendix C in S1 File. These features were identified as key predictors of ADRD and MCI in literature [9, 11, 28] and are presented here to provide insights to the cohorts.

### Model characteristics

Table 2 shows a comparison of the Area Under the Curve (AUC) computed on validation and test data for the best (using grid search) boosted tree, Feed forward network, Recurrent Neural Network and RNN with pre-trained embeddings models for each of the cohorts. The maximum gap between the validation and test datasets for all the models is .6% for year8, while it is less than .3% for all the other models indicating little to no overfitting during the training process. The year0 models perform much better than any of the other models because this uses events over the two-year period leading up to the index date while the other models use events which are farther from the index date. The best AUCs on test data (highlighted) for year8-3 range between 80.7% and 84.4%, with a monotonic improvement from farthest to closest to index date cohorts.

**Table 2:** Area under the curve for validation and test cohorts using different models.

Cohort	Boosted Trees		Feed Forward Network		Recurrent Neural Network		RNN with Pre-trained Embeddings	
	valid	test	valid	test	valid	test	valid	test
year0	92.3%	92.4%	93.0%	93.1%	94.5%	**94.4%**	NA	NA
year3	83.8%	84.1%	84.2%	**84.4%**	84.1%	84.3%	84.1%	84.3%
year4	83.2%	83.3%	83.6%	**83.7%**	83.4%	83.5%	83.5%	83.5%
year5	81.8%	81.8%	82.9%	**83.0%**	82.6%	82.7%	82.7%	82.8%
year6	81.9%	82.1%	82.2%	**82.4%**	81.7%	81.8%	82.0%	82.2%
year7	81.3%	81.1%	81.8%	**81.6%**	81.4%	81.2%	81.8%	81.5%
year8	80.0%	79.9%	81.2%	**80.7%**	77.5%	77.0%	79.7%	79.0%

The Feed Forward Network model does better than the Boosted Tree model for every cohort with differences ranging from 0.3 to 1.2%. It is able to handle the non-linearities and interactions in the data much better than boosted tree model. The FFN also does better than the RNN which encodes the sequence of diagnosis, procedure and drug events into the model for every cohort except year0. This suggests that the sequence of codes leading up to the index date is very important but for periods further from the index date, the codes itself are important but not the order in which they appear. The RNN with pre-trained weights shows the best results from three different embedding weight update strategies discussed earlier. For all the models, the strategy where the weights were frozen to those obtained for year0 was the best even though that meant that the embeddings for several of the codes not seen in year0 but present in other cohorts were set to 0 and hence not used. The models with pre-trained weights performed better than the RNN model (where embedding weights were trained from scratch) by up to 2%.

Table 3 shows the F1 scores computed on validation and test data using the prevalence and thresholds (computation details in Appendix C in S1 File in [28]) from the validation dataset. While the trends discussed for the AUC in terms of superior performance of the Feed Forward Network models across all models and, RNN with pre-trained performing better than RNN, the F1 score for the year0 cohort using RNNs has an absolute difference of 8% from the next best which is clinically significant and reinforces the importance of the sequence of codes.

**Table 3:** F1 scores for validation and test cohorts using different models computed using prevalence in the validation cohort.

Cohort	Boosted Trees		Feed Forward Network		Recurrent Neural Network		RNN with Pre-trained Embeddings	
	validation	test	validation	test	validation	test	validation	test
year0	44.5%	44.6%	46.2%	46.1%	54.0%	**54.1%**	NA	NA
year3	28.5%	29.0%	29.6%	**29.6%**	29.0%	29.0%	29.7%	29.4%
year4	28.4%	28.5%	29.4%	**29.1%**	28.8%	28.5%	29.1%	28.7%
year5	27.4%	27.7%	28.9%	**28.8%**	28.0%	28.1%	28.4%	28.2%
year6	27.6%	27.8%	28.9%	**28.4%**	27.9%	27.0%	27.9%	27.3%
year7	26.9%	26.2%	28.4%	**26.7%**	27.3%	25.9%	28.0%	26.5%
year8	26.0%	25.6%	27.7%	**25.6%**	25.6%	24.5%	26.0%	23.8%

Table 4 shows the training time per epoch for the feed forward network and recurrent neural network models. The train time for the RNN model using LSTMs is much higher (15–50x) than the train time for FFN. The difference in run time is attributed to not just the complexity of an LSTM but also to differences in how the inputs are read; for an FFN the data is stored in the GPU as a tensor 2-dimensional tensor and sliced as required. For the LSTM, since the sequences are of different length, they have to be padded to the size of the longest sequence in a mini-batch and again re-arranged between the embedding and LSTM layers.

**Table 4:** Training time in seconds per epoch for feed-forward network and recurrent neural network models.

Cohort	Features	Feed Forward Network	Recurrent Neural Network
year0	2,772	162.2	4,284.7
year3	1,998	32.4	1,642.4
year4	1,907	25.1	678.4
year5	1,831	15.0	425.4
year6	1,841	8.2	387.9
year7	1,943	8.0	203.1
year8	1,817	3.5	54.0

Extensive grid searches were run for all of the models. They resulted in a 7–11% increase in the test AUC and a 24–36% increase in F1 score across all the models and cohorts. For the boosted tree model the search space was a cartesian of {learning rate: .002, .004, .006, .008} x {number of leaves: 63, 31, 15} x {feature fraction: .02, .05, .1, .2} x {min data in leaf: 300, 600, 1000} with a maximum of 500 boosting rounds and early stopping round limit of 100. For the feed forward network the search space was a cartesian of {learning rate .0002, .0004, .0001}, {batch size 255, 511, 1023} and {hidden layers 2–4, layer widths 31 to 255}. The number of epochs was set to 100 and Adam optimization algorithm [38] was stopped when the binary classification error on the validation data did not improve in 10 iterations. The F1 score was computed using the model score at prevalence as the threshold (example: in a cohort with 100 people of which 5 are cases, the fifth largest model score would be the threshold) and the parameter set which had the largest F1 score on validation data was selected as the grid search winner.

The grid search space for the RNN models was limited because of the long run times; the embedding dimension 75 and LSTM hidden dimension 256 were fixed with 3 variations in fully-connected layers, two choices for learning rate and 3 choices for how the embedding weights should be trained. The fixed choices were set by running ad hoc runs and choosing the parameters with good accuracy with reasonable training times; for example, using an embedding size of 300 resulted in slightly better F1 scores but the training time increase (2x) was not preferred.

## Discussion

The results presented in this work with AUCs ranging from 80% to 85% and F1s from 25 to 30%, 3 to 8 years prior to the index date is much better than any other work using similar type of data. Four models have been trained on each cohort and scored using the validation and test datasets. In all but one of the models, the Feed Forward Network was the best both in terms of AUC which is a global discrimination measure, and F1 score which combines sensitivity and positive predictive value and is computed at a specific threshold. Of the deep learning architectures, the FFN, including the modification suggested in this work, is the easiest to code, and can be trained on a GPU. The model weights can be used to compute the predictions on a CPU and that runs very fast. The results show that the boosted trees are close in terms of accuracy and are worthwhile alternatives.

The bidirectional LSTM model performs very well on the year0 cohort which a F1 score which is 8% better (absolute difference) than the next best model indicating the importance of the sequence of codes very close to the index date. The embeddings from year0 were useful for initializing the embeddings for year3-8. While the results with initialized embeddings were still worse than the FFN, a next step may be to add an attention layer which can help with model interpretability to understand which codes effect the score. The varying data characteristics where closer to the onset the sequences are longer and relative order is important, whereas farther from the onset, the sequences are shorter and the order is not as important must be considered along with the need to compute the attention weights. The additional complexity of the model with only a possible small gain may make the FFN a better option to use in production given the speed and accuracy tradeoff. The importance of age and gender when predicting Alzheimer’s is well known making that a good candidate to add to the last hidden layer so that it is forced into the model. It may not be as obvious for other conditions and attention weights may help identify and quantify such factors.

The choice of grid search parameters both for the boosted tree models and the deep learning models were so that memorization or overfitting could be avoided while still maintaining the accuracy. By increasing the number of leaves or reducing the min data in leaf for the boosted trees or increasing the width and number of the hidden layers or size of the embedding, the test AUC and F1 scores were improved. But in each of those situations, there was a large difference between training and validation and test which was not preferred.

Deep Learning models require larger datasets to perform better than traditional machine learning algorithms. While the FFN model here does indeed perform better as the amount of training data increases from year8 to year3, that improvement may be because the prediction period is closer to the index date and the underlying classification problem is an easier one. The gap for the F1 scores or AUC between FFN and boosted tree models does not correlate with the amount of training data available to each of these models.

## Conclusion

Deep Learning models can be used to build prediction models for ADRD to compute risk scores several years prior to the onset of the disease. While they require more expensive hardware to train models, this can result in large improvements in accuracy where sequences are important. Heterogeneity of early clinical presentation of the condition, captured in thousands of features available in the datasets developed in this work, are synthesized using these models which infer multi-dimensional relationships.

Extensive grid search runs need to be performed to get the best set of parameters for the models. The choice of these parameters is important to prevent overfitting and improve accuracy.

Future work should focus on using the embeddings with deep and wide model or factorization machine-based models which capture interactions. The embeddings can also be clustered and those clusters can be used in place of the original codes which can lead to less sparsity in datasets.

Clinically, an F1 score of 54% at index date should be high enough to allow more efficient screening and pursuit of further patient testing for cognitive decline.

## Supporting information

S1 File (DOCX)
