# Supplementary material for: Deep neural network models for identifying incident dementia using claims and EHR datasets
Source: PLoS One. 2020 Sep 24;15(9):e0236400. doi: 10.1371/journal.pone.0236400 (PMC7514098; doi:10.1371/journal.pone.0236400)
Supplement: S1 File — (DOCX) [file pone.0236400.s001.docx]

# Appendix A: Identification Rules for ADRD and MCI Cases

The identification rules for diagnosed ADRD cases were developed based on past work [11], and extended in consultation with an Expert Advisory Panel consisting of clinicians and experts from academia. Individuals must have met at least one of the following criteria:

1. a medical claim with ADRD (Table A1a) or CI (Table A1b) diagnosis codes in any header position in any setting (inpatient or outpatient) followed by another medical claim with any of these codes between 31 and 540 days,
2. a pharmacy claim for donepezil hydrochloride, galantamine hydrobromide, rivastigmine tartrate or tacrine hydrochloride,
3. a pharmacy claim for memantine hydrochloride along with a medical claim followed by an ADRD or CI diagnosis code in any setting and any header position within 0 to 730 days. The confirmation with a diagnosis claim is required because memantine hydrochloride is also used as an augmentation therapy for anxiety disorders (OCD, ADHD, etc.) [39] as well as help slowing down the tolerance development to opioids [40].
4. a diagnosis code for scans (Table A1c) and a procedure code for scans (Table A1d) on the same day followed by a diagnosis code for ADRD or CI between 7 and 180 days
5. a single failed cognitive test Montreal Cognitive Assessment (<=25), mini-mental state exam (<=23) or Mini-Cog (<=4)

Tables A1a-d. Diagnosis and procedure codes used to identify the cohort.

| ADRD Diagnosis Codes   \|  \| Code \| Description \| \| --- \| --- \| --- \| \| ICD-9 \| 290 \| Dementias \| \| 2900 \| Senile Dementia, Uncomplicated \| \| 29000 \| Alzheimer Senil/Uncmplcd \| \| 2901 \| Presenile Dementia \| \| 29010 \| Presenile Dementia, Uncomplicated \| \| 29011 \| Presenile Dementia With Delirium \| \| 29012 \| Presenile Dementia With Delusional Features \| \| 29013 \| Presenile Dementia With Depressive Features \| \| 2902 \| Senile Dementia With Delusional Or Depressive Features \| \| 29020 \| Senile Dementia With Delusional Features \| \| 29021 \| Senile Dementia With Depressive Features \| \| 2903 \| Senile Dementia With Delirium \| \| 29030 \| Alzheimer Senil/Delirium \| \| 2904 \| Vascular Dementia \| \| 29040 \| Vascular Dementia, Uncomplicated \| \| 29041 \| Vascular Dementia, With Delirium \| \| 29042 \| Vascular Dementia, With Delusions \| \| 29043 \| Vascular Dementia, With Depressed Mood \| \| 2941 \| Dementia In Conditions Classified Elsewhere \| \| 29410 \| Dementia In Conditions Classified Elsewhere Without Behavioral Disturbance \| \| 29411 \| Dementia In Conditions Classified Elsewhere With Behavioral Disturbance \| \| 2942 \| Dementia, Unspecified \| \| 29420 \| Dementia, Unspecified, Without Behavioral Disturbance \| \| 29421 \| Dementia, Unspecified, With Behavioral Disturbance \| \| 2948 \| Other Persistent Mental Disorders Due To Conditions Classified Elsewhere \| \| 29480 \| Organic Mtl/Anxiety Dis \| \| 3310 \| Alzheimer's Disease \| \| 3311 \| Frontotemporal Dementia \| \| 33111 \| Pick's Disease \| \| 33119 \| Other Frontotemporal Dementia \| \| 33182 \| Dementia With Lewy Bodies \| \| 797 \| Senility Without Mention Of Psychosis \| \| ICD-10 \| F0150 \| Vascular Dementia Without Behavioral Disturbance \| \| F0151 \| Vascular Dementia With Behavioral Disturbance \| \| F0280 \| Dementia In Other Diseases Classified Elsewhere Without Behavioral Disturbance \| \| F0281 \| Dementia In Other Diseases Classified Elsewhere With Behavioral Disturbance \| \| F0390 \| Unspecified Dementia Without Behavioral Disturbance \| \| F0391 \| Unspecified Dementia With Behavioral Disturbance \| \| F061 \| Catatonic Disorder Due To Known Physiological Condition \| \| G300 \| Alzheimer's Disease With Early Onset \| \| G301 \| Alzheimer's Disease With Late Onset \| \| G308 \| Other Alzheimer's Disease \| \| G309 \| Alzheimer's Disease, Unspecified \| \| G3101 \| Pick's Disease \| \| G3109 \| Other Frontotemporal Dementia \| \| G3183 \| Dementia With Lewy Bodies \| | CI Diagnosis Codes   \|  \| Code \| Description \| \| --- \| --- \| --- \| \| ICD-9 \| 2940 \| Amnestic Disorder In Conditions Classified Elsew \| \| 29400 \| Amnestic Disorder \| \| 3312 \| Senile Degeneration Of Brain \| \| 33183 \| Mild Cognitive Impairment, So Stated \| \| 78093 \| Memory Loss \| \| ICD-10 \| F04 \| Amnestic Disorder Due To Known Physio Cond \| \| G311 \| Senile Degeneration Of Brain, Not Elsewhere Classified \| \| G3184 \| Mild Cognitive Impairment, So Stated \| \| R411 \| Anterograde Amnesia \| \| R412 \| Retrograde Amnesia \| \| R413 \| Other Amnesia \|   Scan Diagnosis Codes   \|  \| Code \| Description \| \| --- \| --- \| --- \| \| ICD-9 \| 33189 \| Other Cerebral Degeneration \| \| 4380 \| Cognitive Deficits Due To Cerebrovascular Disease \| \| 79952 \| Cognitive Communication Deficit \| \| 79959 \| Other Signs And Symptoms Involving Cognition \| \| ICD-10 \| G3189 \| Other Specified Degen Diseases Of Nervous System \| \| I6991 \| Cognitive Deficits Following Unspecified Cerebrovascular Disease \| \| R4181 \| Age-Related Cognitive Decline \| \| R41841 \| Cognitive Communication Deficit \| \| R4189 \| Other Symptoms And Signs Involving Cognitive Functions And Awareness \|   Scan Procedure Codes   \|  \| Code \| Description \| \| --- \| --- \| --- \| \| CPT \| 3112F \| CT or MRI Of The Brain Performed Greater Than 24 Hours After Arrival To The Hospital Or Performed In An Outpatient \| \| 70450 \| Computed Tomography, Head Or Brain; Without Contrast \| \| 70460 \| Computed Tomography, Head Or Brain; With Contrast \| \| 70470 \| Computed Tomography, Head Or Brain; Without Contrast Material, Followed By Contrast Material(s) And Further Sec \| \| 70551 \| Magnetic Resonance (Eg, Proton) Imaging, Brain (Including Brain Stem); Without Contrast Material \| \| 70552 \| Magnetic Resonance (Eg, Proton) Imaging, Brain (Including Brain Stem); With Contrast Material(s) \| \| 70553 \| Magnetic Resonance (Eg, Proton) Imaging, Brain (Including Brain Stem); Without Contrast Material \| \| IC  D-9 \| 8891 \| Magnetic Resonance Imaging Of Brain And Brain Stem \| \| ICD-10 \| B02000Z \| Computerized Tomography (Ct Scan) Of Brain Using High Osmolar Contrast, Unenhanced And Enhanced \| \| B0200ZZ \| Computerized Tomography (Ct Scan) Of Brain Using High Osmolar Contrast \| \| B02010Z \| Computerized Tomography (Ct Scan) Of Brain Using Low Osmolar Contrast, Unenhanced And Enhanced \| \| B0201ZZ \| Computerized Tomography (CT Scan) Of Brain Using Low Osmolar Contrast \| \| B020Y0Z \| Computerized Tomography (CT Scan) Of Brain Using Other Contrast, Unenhanced And Enhanced \| \| B020YZZ \| Computerized Tomography (CT Scan) Of Brain Using … \| \| B030Y0Z \| Magnetic Resonance Imaging (MRI) Of Brain Using Other Contrast, Unenhanced And Enhanced \| \| C030BZZ \| Positron Emission Tomographic (PET) Imaging Of Brain Using Carbon 11 (C-11) \| \| C030KZZ \| Positron Emission Tomographic (PET) Imaging Of Brain Using Fluorine 18 (F-18) \| \| C030MZZ \| Positron Emission Tomographic (PET) Imaging Of Brain Using Oxygen 15 (O-15) \| \| C030YZZ \| Positron Emission Tomographic (PET) Imaging Of Brain Using Other Radionuclide \| \| HC  PCS \| G0229 \| Pet Imaging, Brain \| \| G0336 \| Pet Imaging, Brain, Alzheimer's \| |
| --- | --- | --- | --- | --- | --- | --- | --- | --- | --- | --- | --- | --- | --- | --- | --- | --- | --- | --- | --- | --- | --- | --- | --- | --- | --- | --- | --- | --- | --- | --- | --- | --- | --- | --- | --- | --- | --- | --- | --- | --- | --- | --- | --- | --- | --- | --- | --- | --- | --- | --- | --- | --- | --- | --- | --- | --- | --- | --- | --- | --- | --- | --- | --- | --- | --- | --- | --- | --- | --- | --- | --- | --- | --- | --- | --- | --- | --- | --- | --- | --- | --- | --- | --- | --- | --- | --- | --- | --- | --- | --- | --- | --- | --- | --- | --- | --- | --- | --- | --- | --- | --- | --- | --- | --- | --- | --- | --- | --- | --- | --- | --- | --- | --- | --- | --- | --- | --- | --- | --- | --- | --- | --- | --- | --- | --- | --- | --- | --- | --- | --- | --- | --- | --- | --- | --- | --- | --- | --- | --- | --- | --- | --- | --- | --- | --- | --- | --- | --- | --- | --- | --- | --- | --- | --- | --- | --- | --- | --- | --- | --- | --- | --- | --- | --- | --- | --- | --- | --- | --- | --- | --- | --- | --- | --- | --- | --- | --- | --- | --- | --- | --- | --- | --- | --- | --- | --- | --- | --- | --- | --- | --- | --- | --- | --- | --- | --- | --- |

# Appendix B: Training and Test Cohorts

The ADRD predictive model was trained and tested using a nested case-control study design [13]. Step-wise demonstration of how the study population was assembled is depicted in Figure B1. The cohorts were constructed using the administrative claims and the structured electronic health record data present in OLDW. This enabled us to create cohorts which were much larger than the published cohorts in [9, 11] for dementia, and could be used for deep learning models which typically perform better with more data [41]. While the claims data contains plan enrollment information which is typically used for establishing continuous enrollment (CE), the EHR data collected from multiple hospital systems does not have this information. To establish a common mechanism for CE, a Technical Experts Panel guiding the cohort development suggested a utilization-based measure where-in a person with at least one fact (claim or diagnosis/procedure in the health record) during a calendar year would be deemed as being continuously enrolled for that year. OLDW contains 42,784,057 unique individuals with at least two years CE and at least 35 years on 1/1/2007.

Out of these individuals 1,824,005 were confirmed cases using the criteria mentioned in the previous section while 31,940,340 do not have any of the cognitive codes listed in Table 1. Applying a two-year CE requirement to ascertain incident dementia and a minimum age requirement on index date, the number of cases reduces to 862,377 and the number of controls to 19,060,279. Using the exclusion criteria described in [26], ignoring individuals who were previously diagnosed cancer, brain diseases, and other serious conditions, and those who have been in long term care facilities for an extended period of time because dementia may be undercoded on those individuals [42, 43] the number of cases drops to 561,093 and controls to 16,666,727. The data is now split in 3 parts – validation (10%), test (20%), and training (70%). While the training dataset is used to train the model, the validation dataset is used to determine the iteration/epoch at which training is stopped, for selecting the best parameter set in the grid search, and for computing the thresholds to dichotomize the score as cases and controls. The test dataset is used to compute the final metrics using the thresholds previously computed.

Seven cohorts labeled year8,…3 and year0 (also called incident cohort), were created each with a 2-year facts collection window which is 8, 7, 6, 5, 4, 3 and 0 years before the index date. The following description will focus on the cohort which collects facts during the sixth and seventh year (X=5) prior to the index date. Requiring CE during the 6^th^ and 7^th^ years, and that the age be at least 45 years on the facts collection end date, drops the number of cases for train / validation / test to 69,598 / 9,984 / 20,338 and the number of controls to 966,416 / 138,388 / 276,095. The counts for the other cohorts are seen in Figure B1.

| 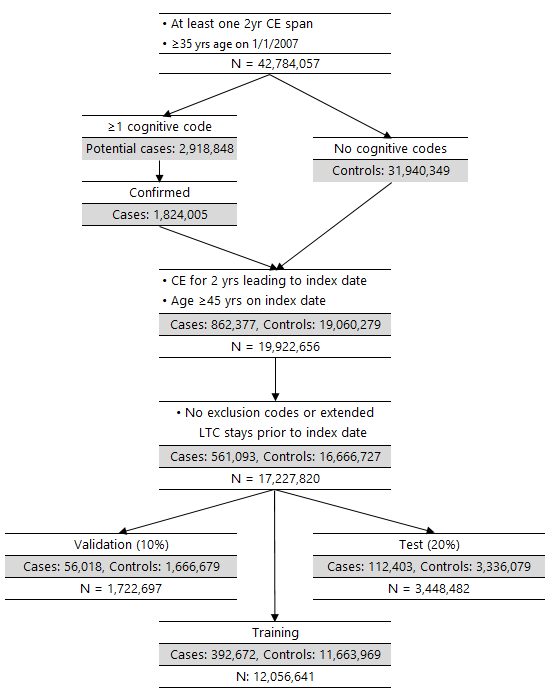 | 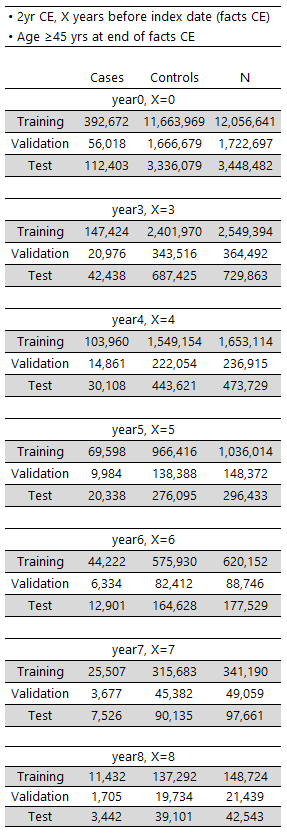 |
| --- | --- |

Figure B1: Study design with training, validation and test cohorts.

# Appendix C: Top Codes for Each Cohort

The univariate odds ratios of some of the diagnosis, procedure and drug features out of thousands of features in the training cohorts with a prevalence of at least 1% in either the cases or controls are presented in Table C1. These features were identified as key predictors of ADRD and MCI in literature [9, 11, 26] and are presented here to provide insights to the cohorts; all the features (and not just the ones here) are available to the models during the training. The diagnoses and drugs have been presented at an aggregate level because the individual codes (ICDs or generic drug names) were very sparse. Almost all the features show monotonic reduction in odds ratios for cohorts as facts are collected away from the index date. Some of the features are only present in a few of the cohorts and not in the others.

Table C1.: Univariate odds ratios for important clinical features in training cohorts. Blank indicates that the feature is not present in the cohort for that year.

| Type | Code | Description | year0 | year3 | year4 | year5 | year6 | year7 | year8 |
| --- | --- | --- | --- | --- | --- | --- | --- | --- | --- |
| Drugs | | Dopamine Precursors | 11.65 | 7.11 | 6.30 |  |  |  |  |
|  |  | Butyrophenones | 6.87 |  |  |  |  |  |  |
|  |  | Atypical Antipsychotics | 5.03 | 2.34 | 2.28 | 2.20 | 2.08 | 1.93 |  |
|  |  | Antimuscarinics | 3.94 | 2.92 | 2.76 | 2.64 | 2.56 | 2.32 | 2.25 |
|  |  | Non-ergot Derived Dopamine Receptor Agonist | 3.02 | 2.25 | 2.24 | 2.22 | 2.13 | 2.06 | 2.16 |
|  |  | Serotonin Modulators | 2.42 | 1.70 | 1.67 | 1.63 | 1.56 | 1.66 | 1.50 |
|  |  | Selective-serotonin Reuptake Inhibitors | 2.41 | 1.50 | 1.43 | 1.38 | 1.34 | 1.33 | 1.30 |
|  |  | Antidepressants, Miscellaneous | 2.36 | 1.39 | 1.34 | 1.27 | 1.24 | 1.24 | 1.18 |
|  |  | Selective Serotonin Norepinephrine Reuptake Inh | 1.89 | 1.44 | 1.42 | 1.38 | 1.32 | 1.37 | 1.26 |
|  |  | Tricyclics, Other Norepi-ru Inhibitors | 1.89 | 1.63 | 1.61 | 1.54 | 1.56 | 1.52 | 1.52 |
| Proce- dures | 96119 | Neuropsychological Testing - Admin by Physician | 120.33 |  |  |  |  |  |  |
|  | 96118 | Neuropsychological Testing - Admin by Technician | 103.27 |  |  |  |  |  |  |
|  | 96116 | Neurobehavioral Status Exam | 82.29 |  |  |  |  |  |  |
|  | 70551 | Magnetic Resonance Imaging | 9.84 | 3.07 | 2.97 | 2.80 | 2.64 | 2.37 | 2.24 |
|  | 70450 | Computed Tomography, Head Or Brain (no Contrast) | 7.44 | 3.30 | 3.07 | 2.82 | 2.60 | 2.36 | 2.18 |
|  | 70470 | Computed Tomography, Head Or Brain | 6.39 |  |  |  |  |  |  |
|  | 70553 | Magnetic Resonance Imaging | 4.82 | 2.14 | 2.04 | 1.88 | 1.77 | 1.81 | 1.63 |
|  | 90801 | Psychiatric Diagnostic Interview Examination | 2.48 | 1.26 | 1.23 | 1.18 | 1.23 | 1.07 | 0.91 |
|  | 90862 | Pharmacological Management | 1.93 | 1.52 | 1.53 | 1.50 | 1.52 | 1.47 | 1.32 |
|  | 90805 | Individual Psychotherapy |  | 1.68 | 1.73 | 1.77 | 1.87 | 1.66 | 1.42 |
| Diagnoses | | Parkinson's Disease | 15.07 | 10.54 | 10.11 | 10.11 | 9.60 | 8.56 |  |
|  |  | Mental Disorders, Organic & Drug-induced | 14.42 | 4.34 | 3.99 | 3.52 |  |  |  |
|  |  | Psychotic & Schizophrenic Disorders | 13.84 | 4.36 | 3.82 | 3.63 | 3.24 |  |  |
|  |  | Hereditary & Degenerative Diseases Of Ce | 9.13 | 3.72 | 3.37 | 3.29 | 3.05 | 2.83 | 2.52 |
|  |  | Neurological Diseases Signs & Symptoms | 6.02 | 2.61 | 2.45 | 2.31 | 2.20 | 2.02 | 1.76 |
|  |  | Cerebral Vascular Disease | 5.97 | 3.46 | 3.38 | 3.28 | 3.20 | 3.05 | 2.91 |
|  |  | Brain Trauma | 5.28 | 2.78 | 2.66 | 2.57 |  |  |  |
|  |  | Psychiatric Diseases Signs & Symptoms | 4.87 |  |  |  |  |  |  |
|  |  | Congenital Disorders Of Central Nervous | 4.67 | 2.94 | 2.89 | 2.84 | 2.57 | 2.50 | 2.06 |
|  |  | Acute Renal Failure | 4.02 | 2.92 | 2.86 | 2.66 | 2.80 | 2.69 |  |

# References

[39] Zdanys K, Tampi RR. A systematic review of off-label uses of memantine for psychiatric disorders. Prog Neuropsychopharmacol Biol Psychiatry. 2008;32(6):1362-1374. doi:10.1016/j.pnpbp.2008.01.008

[40] Grande LA, O’Donnell BR, Fitzgibbon DR, Terman GW. Ultra-low dose ketamine and memantine treatment for pain in an opioid-tolerant oncology patient. Anesth Analg. 2008;107(4):1380-1383. doi:10.1213/ane.0b013e3181733ddd

[41] Chen D, Liu S, Kingsbury P, et al. Deep learning and alternative learning strategies for retrospective real-world clinical data. npj Digital Medicine. 2019;2(1):1-5. doi:10.1038/s41746-019-0122-0

[42] Boustani M, Zimmerman S, Williams CS, et al. Characteristics associated with behavioral symptoms related to dementia in long-term care residents. Gerontologist. 2005;45 Spec No 1(1):56-61. doi:10.1093/geront/45.suppl_1.56

[43] Smith M, Buckwalter KC, Kang H, Ellingrod V, Schultz SK. Dementia care in assisted living: needs and challenges. Issues Ment Health Nurs. 2008;29(8):817-838. doi:10.1080/01612840802182839
